# Inhibitors of glycosomal protein import provide new leads against trypanosomiasis

**DOI:** 10.15698/mic2017.07.581

**Published:** 2017-07-03

**Authors:** Vishal C. Kalel, Leonidas Emmanouilidis, Maciej Dawidowski, Wolfgang Schliebs, Michael Sattler, Grzegorz M. Popowicz, Ralf Erdmann

**Affiliations:** 1 Institute of Biochemistry and Pathobiochemistry, Department of Systems Biochemistry, Faculty of Medicine, Ruhr University Bochum, 44780 Bochum, Germany.; 2 Institute of Structural Biology, Helmholtz Zentrum München, Ingolstädter Landstr. 1, 85764 Neuherberg, Germany.; 3 Center for Integrated Protein Science Munich at Chair of Biomolecular NMR, Department Chemie, Technische Universität München, Lichtenbergstr. 4, 85747 Garching, Germany.; 4 Chair and Department of Drug Technology and Pharmaceutical Biotechnology, Medical University of Warsaw, Banacha 1, 02-097 Warszawa, Poland.

**Keywords:** Trypanosoma, glycosomes, PEX, protein-protein interactions, small molecule inhibitors

## Abstract

Vector-borne trypanosomatid parasite infections in tropical and sub-tropical countries constitute a major threat to humans and livestock. *Trypanosoma brucei* parasites are transmitted by tsetse fly and lead to African sleeping sickness in humans and Nagana in cattle. In Latin American countries, *Trypanosoma cruzi* infections spread by triatomine kissing bugs lead to Chagas disease. Various species of *Leishmania* transmitted to humans by phlebotomine sandflies manifest in a spectrum of diseases termed Leishmaniasis. 20 million people are currently infected with trypanosomatid parasites, leading to over 30,000 deaths annually and half billion people at risk of the infection. It is estimated that 300,000 Chagas infected people reside in the United States and 100,000 in Europe. Glycosomes are peroxisome-like organelles found only in trypanosomatids. Glycolysis occurs in the cytosol in all other organisms, but glycolytic enzymes and other metabolic pathways are compartmentalized inside glycosomes in trypanosomatids. Glycosomes are essential for the parasite survival and hence thought to be an attractive drug target. Our recent study [Dawidowski *et al.* Science (2017)] is the first to report small molecule inhibitors of glycosomal protein import. Using structure-based drug design, we developed small molecule inhibitors of the *Trypanosoma* PEX5-PEX14 protein-protein interaction that disrupt glycosomal protein import and kill the parasites. Oral treatment of *T. brucei* infected mice with PEX14 inhibitor significantly reduced the parasite levels with no adverse effect on mice. The study provides the grounds for further development of the glycosome inhibitors into clinical candidates and validates the parasite protein-protein interactions as drug targets.

Neglected tropical diseases (NTDs) constitute a group of 17 infectious diseases, which mostly affect developing countries and therefore receive less attention for development of new, better, and affordable therapies. African sleeping sickness and kala-azar (visceral Leishmaniasis) are fatal if untreated. Chronic Chagas infections result in cardiomyopathy and may lead to death due to heart failure. Current medications have several limitations such as severe side effects, require complicated treatment schedule, and lack efficacy against different stages of the disease or the strains of the parasites. Emergence of drug resistance is also a major concern. These parasites employ several immune evasion strategies such as antigenic variation; therefore no vaccines are available. Thus, identification of new drug targets and effective treatments is urgently needed.

Glycosomes are unique organelles of the trypanosomatid parasites that are essential for their survival. The first seven enzymes of glycolysis are sequestered inside the glycosomes. Glycolytic enzymes of these parasites such as hexokinase and phosphofructokinase lack feedback-regulation. Sequestering these enzymes in the lumen of glycosomes confers a spatial control over their activities since glycosomal ATP content is limited. There is no net ATP production inside glycosomes; the glycolytic enzymes that produce net ATP are localized in the cytosol. Genetic studies have shown that disruption of glycosomal targeting of the glycosomal enzymes is lethal for the parasites. Mislocalisation of the glycosomal enzymes to the cytosol results in runaway glucose phosphorylation, which accumulates glucose metabolites to toxic levels and concomitantly depletes cellular ATP levels thereby killing the parasites. Glucose becomes toxic to the parasites when glycosomal protein import is defective. Glycosomes also harbor other essential metabolic pathways such as gluconeogenesis, purine salvage, pyrimidine biosynthesis, sugar-nucleotide biosynthesis, pentose phosphate pathway, and oxidative stress defense. A general disruption of glycosomal metabolic pathways as caused by inhibition of glycosomal protein import therefore would also have deleterious effect on the parasites in glucose limiting niches such as intracellular amastigote stage.

Glycosome biogenesis requires the concerted action of Peroxin (PEX) proteins (Fig. 1). Glycosomal enzymes contain peroxisome targeting signals such as PTS1 (conserved C-terminal tripeptide) or PTS2 (nonapeptide near N-terminus). Upon their synthesis in the cytosol, the glycosomal enzymes are recognized by the cytosolic receptors PEX5 or PEX7. The C-terminal tetratricopeptide repeat (TPR) domain of PEX5 recognizes PTS1, while the WD40 repeat containing PEX7 recognizes PTS2 cargo proteins. Trypanosomal PEX5 contains a PEX7 binding region; therefore glycosomal import of both PTS1 and PTS2 proteins depends on PEX5. The cargo loaded receptors dock at the glycosomal membrane by binding to PEX14. PEX5-PEX14 binding results in the formation of a dynamic transient import pore, which allows translocation of the enzymes into the glycosomal lumen. The intrinsically disordered N-terminal part of PEX5 contains several diaromatic pentapeptide motifs (consensus sequence WxxxF/Y), which bind to the small globular N-terminal domain of PEX14. This interaction is essential for glycosomal protein import, therefore a potential drug target against trypanosomatids.

**Figure 1:**
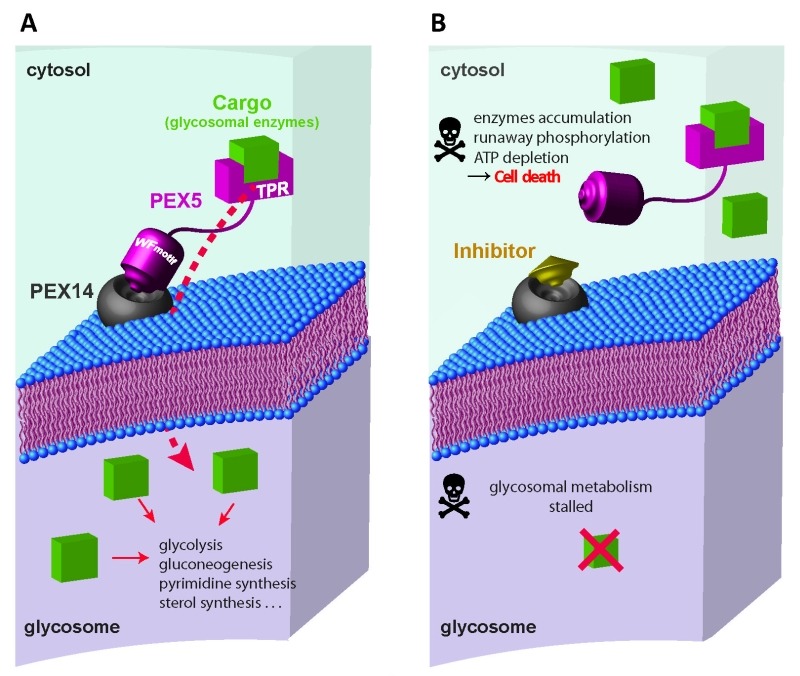
FIGURE 1: Glycosomal protein import as drug target. **(A)** Glycosomal enzymes synthesized in the cytosol are recognized by receptors PEX5 or PEX7, and targeted to glycosomes. C-terminal region of PEX5 contains tetratricopeptide repeat (TPR) domains which recognize the cargo proteins containing PTS1 signal. The cargo-loaded receptor docks at the glycosomal membrane through interaction of PEX5 with PEX14. N-terminal region of PEX5 contains diaromatic pentapeptide motifs (WxxxF/Y), which bind to N-terminal domain of PEX14. This interaction forms a transient pore in the glycosomal membrane that allows import of the enzymes into the glycosomal lumen. **(B) **The inhibitors of PEX5-PEX14 interaction disrupt glycosomal protein import and mislocalise glycosomal enzymes into the cytosol. Uncontrolled activities of the mislocalised glycolytic enzymes cause runaway glucose phosphorylation which accumulates glucose metabolites to toxic levels, depletes cellular ATP levels and the metabolic imbalance kills the parasite.

For structure-based drug design of inhibitors of the PEX5-PEX14 interaction, first the structure of the PEX5-binding domain of *Trypanosoma* PEX14 was determined using nuclear magnetic resonance (NMR), which in combination with other structural information revealed the architecture of PEX5 binding interface in PEX14. The aromatic residues of PEX5 WxxxF/Y motif are accommodated in two hydrophobic pockets flanking the central part of the binding interface in PEX14 (Fig. 2A). To mimic the binding of PEX5 motifs to PEX14, a 3D-pharmacophore model (Fig. 2B) was generated and applied to perform an *in silico* screening of the ZINC library of commercially available 21 million compounds followed by 3D docking. PEX14-binding hits identified *in silico* were further tested and validated by NMR binding assays, monitoring spectral changes of the protein, which led to identification of the drug-like pyrazolo[4,3-c]pyridine molecule. This compound exhibited a moderate affinity to PEX14 and AlphaScreen-based competition assays confirmed that it can inhibit the PEX5-PEX14 interaction *in vitro*. This compound also showed promising anti-trypanosomal activity when tested against cultured bloodstream form of *T. brucei brucei* (which cause Nagana in cattle).

**Figure 2:**
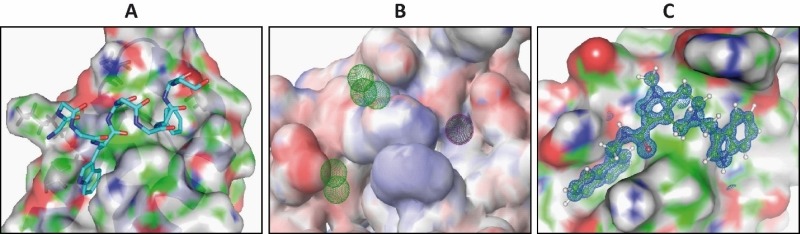
FIGURE 2: Structure based design of the inhibitors of PEX5-PEX14 interaction. **(A)** Structure of *Trypanosoma* PEX14 N-terminal domain bound to PEX5 diaromatic pentapeptide motif. **(B)** 3D-Pharmacophore model generated on the basis of the structure. Spatial placements of hydrophobic moieties were defined as spheres on protein surface. **(C)** X-ray crystal structure of inhibitor bound *Trypanosoma* PEX14. The molecule satisfies pharmacophore model and is able to outcompete PEX5 from PEX14 binding interface.

To optimize the initial compound, an NMR-based fragment screen identified fragment motifs that favorably bind to *Trypanosoma* PEX14. The identified PEX14-binding fragments were used to decorate the initial compound, which yielded new molecules with higher affinity to PEX14 and enhanced trypanocidal activity. After additional medicinal chemistry optimization, a potent and selective *Trypanosoma* PEX5-PEX14 interaction inhibitor was generated. This molecule had low nanomolar trypanocidal activity against cultured bloodstream form of human pathogenic *T. brucei rhodesiense* (which causes African sleeping sickness). The NMR assay data also indicated that the new compound also binds to *T. cruzi* PEX14. When tested against *T. cruzi* amastigotes (the intracellular stage inside cultured human myoblast host cells), PEX14 inhibitor showed a two-fold higher trypanocidal activity than the currently used drug Benznidazole.

The *in vitro* PEX5-PEX14 interaction inhibitory activities of the compounds (Ki) correlate well with the observed anti-trypanosomal activities (IC_50_), indicating that the compounds in the parasites act on-target. High-resolution X-ray crystal structures of the inhibitor bound *Trypanosoma* PEX14 showed that the inhibitors occupy the PEX5-binding site in PEX14 (Fig. 2C). Treatment of cultured *T. brucei* parasites with PEX14 inhibitor led to mislocalisation of glycosomal enzymes to the cytosol. PTS1 and PTS2 containing glycolytic enzymes, respectively phosphofructokinase and hexokinase, were mislocalised to the cytosol. As these enzymes lack feedback-regulation, their mislocalisation to the cytosol results in uncontrolled glucose phosphorylation, which depleted the cellular ATP levels and killed the parasites. Previous PEX14 RNAi-knockdown studies had shown that glucose becomes toxic to glycosome defective trypanosomes. Accordingly, the PEX14 inhibitors were significantly more toxic to trypanosomes when the parasites were grown in glucose rich media. This is due to the fact that already minute amounts of mislocalised glycosomal enzymes are known to disrupt the corresponding metabolic pathways, which thus amplifies the toxic effect on glucose-grown trypanosomes. Accordingly, it was observed that the trypanocidal activities of the compounds were several folds higher than the *in vitro* PEX5-PEX14 inhibition.

For the evaluation of therapeutic potential of PEX14 inhibitors* in vivo*, a bioluminescent animal model of African sleeping sickness was applied. However, due to high plasma protein binding, the molecule used for *in vitro* studies did not affect the parasitemia significantly. Further optimization of the inhibitor yielded another compound exhibiting reduced plasma protein binding, which increased the concentration of free PEX14 inhibitor available in the bloodstream. Oral treatment of *T. brucei *infected mice (twice a day for 5 days) with this molecule led to significant reduction in the parasitemia comparable to the reference drug Suramin.

Glycosome function and biogenesis have long been proposed as attractive drug targets, and inhibitors of glycosomal enzymes have been reported before. The study reviewed here is the first to report small molecule inhibitors of the essential PEX5-PEX14 interaction, which results in disruption of all glycosomal metabolic pathways, thus achieving a multi-pronged and efficient trypanocidal effect. The report provided the structural basis that will facilitate further development of PEX14 inhibitors into clinical candidates and confirms that essential protein-protein interactions of the parasites are potential drug targets and druggable.

